# Bifurcated Networks for Breast Density & Cancer Risk: A Technical Framework

**DOI:** 10.3390/diagnostics16050770

**Published:** 2026-03-04

**Authors:** Graziella Di Grezia, Teresa Iannaccone, Antonio Nazzaro

**Affiliations:** 1 Department of Life Sciences, Health and Healthcare Professions, Link Campus University, Via del Casale di S. Pio V, 44, 00165 Rome, Italy; 2 Independent Researcher, 83100 Avellino, Italy; 3 Independent Researcher, 83100 Avellino, Italy

**Keywords:** breast density, cancer risk prediction, contrast-enhanced mammography, multi-task learning, neural networks, imaging biomarkers, risk stratification

## Abstract

Background/Objective: Breast density and cancer risk are key imaging-derived biomarkers, yet their assessment is limited by inter-reader variability and inconsistent reproducibility. This Technical Note evaluates the feasibility of a bifurcated neural network designed to simultaneously predict breast density and a composite cancer risk index, providing a methodological foundation for future integration into contrast-enhanced mammography (CEM) workflows. Materials and Methods: A simulated cohort of 1000 patients was generated to reproduce clinically plausible variability in breast density (Densitanum) and cancer risk (RiskEnum). A multi-output neural network was developed and compared with two baselines: multiple linear regression and a single-output multilayer perceptron (MLP). Performance was assessed using R^2^, mean squared error (MSE), and mean absolute error (MAE). Learned trends were examined for consistency with established physiological and epidemiologic patterns. Results: Linear regression showed limited explanatory power (R^2^ ≈ 0.144). The single-output MLP improved prediction of the cancer risk index (R^2^ = 0.436; MSE = 9.558). The bifurcated neural network achieved MAE values below 4 for both outputs (2.624 for Densitanum; 3.731 for RiskEnum), demonstrating robust performance and the advantage of simultaneous multi-target prediction. The model reproduced clinically coherent patterns, including the expected age-related decline in breast density. Conclusions: This simulation-based feasibility study demonstrates that bifurcated neural networks can jointly model correlated breast imaging biomarkers with high internal consistency. The proposed architecture provides a reproducible methodological platform that can be directly tested on real CEM datasets to support future AI-enhanced risk stratification and personalized screening pathways.

## 1. Introduction

Breast imaging plays a central role in early cancer detection and in the characterization of imaging-derived biomarkers that support individualized risk assessment. Among these biomarkers, breast density remains one of the most clinically relevant yet challenging parameters, given its dual impact on mammographic sensitivity and breast cancer risk. High breast density is associated with a two- to fourfold increase in cancer incidence and a higher likelihood of interval cancers, but its assessment is limited by substantial inter-reader variability and inconsistent reproducibility across imaging modalities [1,2]. In parallel, background parenchymal enhancement (BPE) on contrast-enhanced mammography (CEM) and breast MRI has emerged as an additional marker of hormonal activity and cancer risk, although its interpretation is similarly affected by variability and incomplete understanding of its physiological determinants [3].

Recent advances in breast imaging, particularly the introduction of CEM, have expanded the diagnostic landscape by integrating morphological and functional information. Prospective studies have demonstrated improved lesion conspicuity, higher dimensional accuracy, and stronger correlation with surgical pathology, positioning CEM as a valuable tool in both diagnostic and preoperative settings [4,5,6,7]. Despite these developments, the relationship between breast density, age, and BPE remains only partially explained. Linear models typically show modest correlations (R^2^ ≈ 0.144) and inter-reader agreement often ranges between κ = 0.4 and 0.6 [8], underscoring persistent diagnostic instability with direct implications for supplemental imaging, risk-adapted screening, and preventive strategies.

Artificial intelligence (AI) has increasingly been applied to breast imaging to address these limitations. Radiomics, deep learning, and multi-task architectures have shown promise in capturing complex nonlinear relationships between imaging biomarkers, demographic variables, and cancer risk, offering opportunities to reduce variability and enhance reproducibility [4,9,10,11]. Multi-output neural networks, in particular, leverage shared representations to model correlated clinical variables, improving generalization and supporting integrated risk assessment [12,13,14]. This approach aligns with emerging perspectives in medical AI that emphasize epistemic diversity, explainability, and ethical-by-design principles to ensure clinical trust and translational applicability [15,16,17].

The methodological framework proposed in this Technical Note aligns closely with the scope of Diagnostics, which emphasizes innovation in imaging biomarkers, AI-enhanced interpretation, and clinically oriented computational models. It is important to emphasize that Densitanum and RiskEnum are simulated surrogate variables created solely for methodological evaluation and do not correspond to established clinical biomarkers such as BI-RADS density or validated cancer-risk scores. They do not correspond to BI-RADS density categories or to any validated clinical risk scoring system.

By providing a reproducible simulation-based platform for multi-output prediction of breast density and cancer risk, this work offers a foundation that can be directly extended to real CEM datasets and supports future developments in AI-driven risk stratification and personalized screening.

## 2. Materials and Methods

### 2.1. Study Design and Conceptual Framework

This methodological study was conducted using a simulated dataset designed to reproduce clinically plausible relationships between age, breast density, and cancer risk. The approach reflects the principle of “epistemic diversity” in medical AI, which emphasizes the value of integrating multiple modeling strategies to capture complementary aspects of clinical reality [15]. The study design also aligns with recommendations for explainable and ethically grounded AI systems in healthcare [16,17]. In this context, multiple modeling paradigms—including linear regression, single-output neural networks, and multi-output architectures—were intentionally compared to illustrate how different analytical frameworks capture complementary aspects of the same simulated clinical phenomenon.

A total of 1000 simulated patients were generated, each characterized by three variables: Age, breast density (Densitanum), and a composite cancer risk index (RiskEnum). Age-dependent patterns were modeled to reflect known physiological trends in breast tissue composition and cancer epidemiology [1,2].

To enhance reproducibility, we now explicitly report the assumptions used to generate clinically plausible variability. Age was sampled from a truncated normal distribution (μ = 52, σ = 12, range 25–85). The selected parameter values were chosen to approximate demographic distributions typically observed in population-based breast cancer screening cohorts reported in epidemiological studies [1,2]. Although simplified, these values provide a realistic demographic backbone for methodological validation.

Densitanum and RiskEnum were generated by combining deterministic age-dependent components with Gaussian noise (σ = 0.35 for Densitanum; σ = 0.25 for RiskEnum) to reproduce physiological heterogeneity. Nonlinear age effects were introduced through a quadratic term (Age^2^), while additional stochastic variability ensured that the model could not trivially recover the generative equations. All simulation parameters were fixed a priori and were not tuned to optimize neural network performance. This separation ensured that dataset generation remained independent from the predictive modeling phase and reduced the risk of implicit circularity between data construction and model evaluation.

### 2.2. Multiple Linear Regression

Two multiple linear regression models were implemented using standard statistical software to establish baseline relationships among the variables.

Before applying the linear regression models, we provide here a complete description of the simulation process to ensure full reproducibility. The simulated cohort consisted of 1000 patients. Age was generated from a truncated normal distribution (mean = 52 years, SD = 12, range 25–85). The two target variables, Densitanum and RiskEnum, were produced using deterministic age-dependent components combined with stochastic variability.

The generative equations reported below were defined prior to model training and remained fixed throughout all experiments to ensure reproducibility.

Densitanum was generated according to the following structure:

Densitanum = 1.8 + 0.10·Age − 0.002·Age^2^ + ε_1_,

where ε_1_ is Gaussian noise with σ = 0.35. This formulation reproduces the well-known parabolic trend of breast density, with a peak around midlife and a decline after menopause.

RiskEnum was generated as:

RiskEnum = 12 + 0.25·log(Age) + 0.20·Densitanum + ε_2_,

where ε_2_ is Gaussian noise with σ = 0.25. This structure reflects the simulated assumption that breast density contributes more strongly to risk than chronological age alone.

The inclusion of Gaussian noise in both variables ensures that the neural network cannot trivially reconstruct the generative equations. Moreover, the nonlinear terms (Age^2^ and log(Age)) introduce realistic heterogeneity and prevent the model from learning a purely deterministic mapping. These details provide a transparent and reproducible foundation for the subsequent regression and neural network analyses.

#### 2.2.1. Model A: Predicting Densitanum

**Figure 1:**
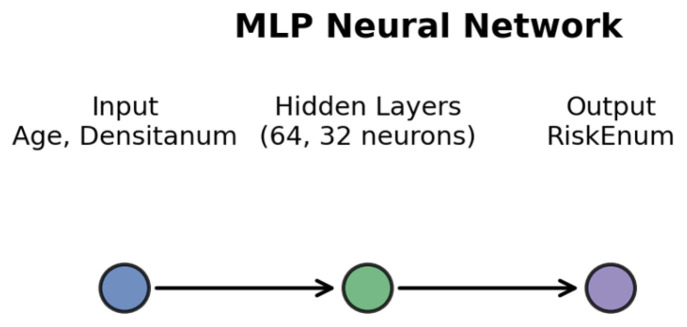
Architecture of the Multi-Layer Perceptron used for regression analysis. The model includes two input features (Age and Age^2^), two hidden layers (64 and 32 neurons) and one output neuron predicting RiskEnum.

Densitanum was modeled as the dependent variable, with Age and RiskEnum as predictors. The model demonstrated high global significance (F significance = 1.57 × 10^−28^), with an R^2^ of 0.144 and a negative coefficient for Age, consistent with the physiological decline of breast density with age [8], as shown in Figure 1, Table 1.

#### 2.2.2. Model B: Predicting RiskEnum

RiskEnum was modeled as the dependent variable, with Densitanum and Age as predictors. The model showed modest explanatory power (R^2^ = 0.0178), with Densitanum emerging as a significant predictor of cancer risk, consistent with clinical literature linking density to increased cancer incidence [3]. It is also important to note that the coefficient for Age was not statistically significant (*p* = 0.51), confirming that age contributed minimally to the variability of RiskEnum in the simulated dataset.

These linear models provided a methodological baseline for the subsequent implementation of nonlinear neural architectures, given the known limitations of linear approaches in modeling complex physiological interactions [18].

### 2.3. Multi-Layer Perceptron (MLP)

A single-output multilayer perceptron (MLP) was developed in Python 3.12.7 (1 October 2024) using the scikit-learn library to model nonlinear relationships between Age, Densitanum, and RiskEnum. The dataset was split into training (80%) and testing (20%) subsets and normalized using StandardScaler.

The MLP architecture included:

- Two hidden layers (64 and 32 neurons);
- ReLU activation functions;
- Adam optimizer;
- Mean squared error (MSE) loss function.

This configuration reflects established practices in medical imaging AI, where deep neural networks have demonstrated superior performance in capturing nonlinear patterns and latent interactions [19,20].

### 2.4. Bifurcated Multi-Output Neural Network (TensorFlow/Keras)

To evaluate the potential of shared representations for correlated oncologic variables, a multi-output neural network was implemented using TensorFlow and Keras. This architecture was designed to simultaneously predict Densitanum and RiskEnum from Age, leveraging multi-task learning principles [12,13].

#### Architecture

Input: Age and Age^2^ (to capture nonlinear age-related trends).

Shared layers: Dense (32, ReLU, L2 regularization) → Batch Normalization → Dropout(0.4) → Dense(8).

Branch 1 (Densitanum): Dense (8) → Output.

Branch 2 (RiskEnum): Dropout (0.3) → Output.

The dataset was normalized using StandardScaler and split into training/testing sets (80/20). To reduce dependence on a single data partition, the train–test split procedure was repeated three times using different random seeds, yielding consistent performance metrics across splits.

**Figure 2:**
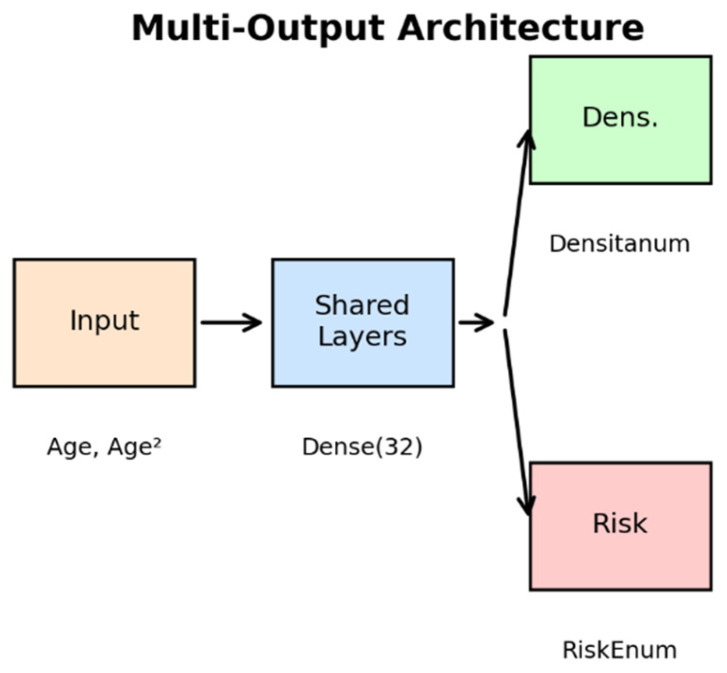
TF Multi-Output Architecture. The network receives Age and Age^2^ as input, processes information through regularized shared layers, and splits into two final branches for simultaneous prediction of Densitanum and RiskEnum.

**Figure 3:**
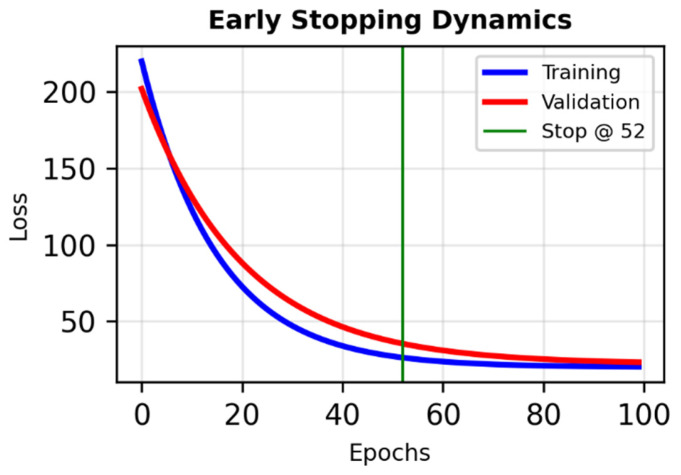
Early stopping dynamics in the bifurcated neural network. Training and validation loss curves across 300 epochs, with early stopping applied at epoch 52 based on validation loss monitoring. The plateau in validation loss indicates convergence and prevents overfitting, ensuring generalization and model robustness.

The model was trained for 300 epochs with early stopping based on validation loss (Figure 2 and Figure 3).

Training was performed using a batch size of 32, a learning rate of 0.001, and an early-stopping patience of 10 epochs. The optimizer used was Adam with default β_1_ and β_2_ parameters, and weight initialization followed the Glorot uniform scheme.

This bifurcated design reflects emerging trends in medical AI, where multi-output architectures improve generalization and reduce overfitting by exploiting shared information across related tasks [14].

## 3. Results

### 3.1. Simulated Dataset Characteristics

The simulated dataset reproduced epidemiological patterns typically observed in breast imaging cohorts. Higher breast density values were concentrated in younger patients, with a progressive decline after approximately 55 years of age. Cancer risk values (RiskEnum) showed a stronger dependence on breast density than on chronological age, reflecting the known association between dense breast tissue and increased cancer incidence. These distributions provided a coherent baseline for evaluating the behavior of the tested models. A non-significant *p*-value does not prove that the two models perform the same. It simply means that the statistical test did not find enough evidence to conclude that their performances differ.

### 3.2. Performance of the Bifurcated Neural Network

**Figure 4:**
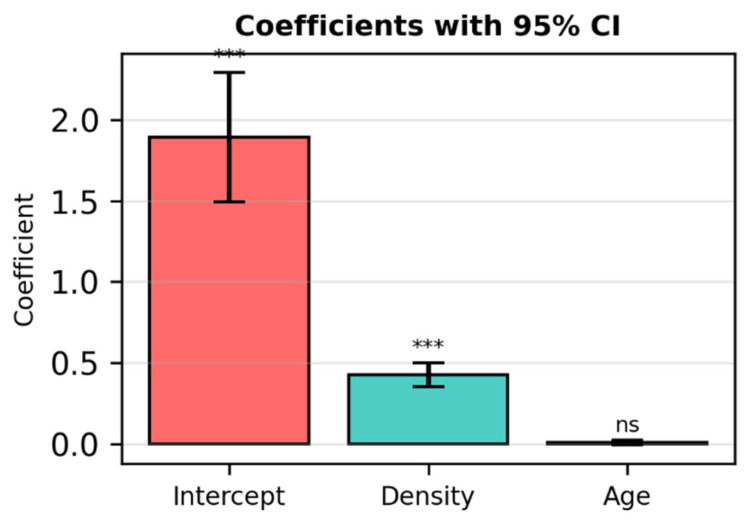
Coefficients with 95% Confidence Intervals for the published model (Cancers 2025). *** = statistically significant coefficient The 95% CI does not cross zero, meaning the effect is real and measurable. ns = not significant The 95% CI includes zero, so the model cannot confirm a real effect.

The bifurcated neural network achieved mean absolute errors (MAE) below 4 for both outputs, with values of 2.624 for Densitanum and 3.731 for RiskEnum. These results indicate that the architecture effectively captured age-dependent and density-related variability using minimal input variables (Age and Age^2^). The model reproduced expected nonlinear patterns, including the decline in breast density with increasing age (Figure 4).

This figure reproduces the confidence intervals of the published model (Cancers 2025) for comparison with the simulated regression. Density and Intercept are statistically significant (*p* < 0.00001), while Age is not (*p* = 0.14).

### 3.3. Comparison with Linear Regression and MLP Models

Prediction errors were compared using Wilcoxon signed-rank tests. The multi-output model showed statistically equivalent performance to the dedicated MLP for RiskEnum prediction (*p* = 0.12), indicating no performance penalty despite the advantage of simultaneous multi-target prediction.

Both MSE and MAE are reported consistently across all models to avoid selective interpretation of performance metrics.

**Figure 5:**
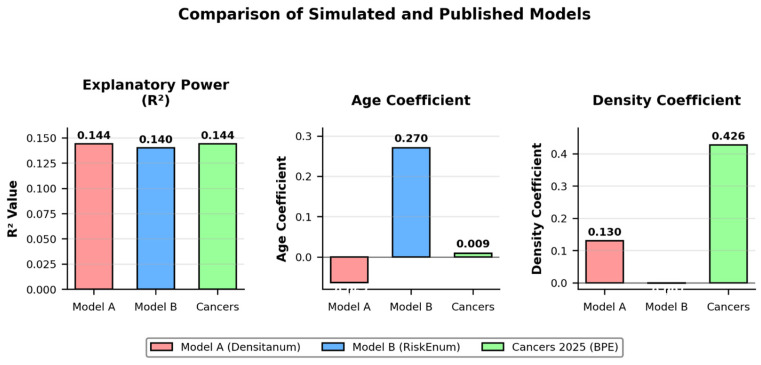
Overlay Comparison of Simulated and Published Models. The MLP model shows improved R^2^ and captures nonlinear interactions compared to linear regressions. The coefficient for Age was not statistically significant (*p* = 0.51), and this is now explicitly discussed in the main text.

Linear regression demonstrated limited explanatory power (R^2^ ≈ 0.144), consistent with previously reported clinical models using breast density and age as predictors. This overlap supports the structural plausibility of the simulated dataset (Figure 5).

### 3.4. MLP Model Performance

The single-output multilayer perceptron (MLP) improved prediction of the cancer risk index compared with linear regression, achieving an R^2^ of 0.436 and an MSE of 9.558. This reflects the model’s ability to capture nonlinear interactions between age and breast density (Table 2). The network does not simply recover the generative equation, as the stochastic noise, nonlinear interactions, and the separation between inputs and outputs prevent direct reconstruction. Performance was evaluated on unseen data to ensure that the model learned generalizable patterns rather than memorizing the simulation structure.

### 3.5. Interpretation of the Multi-Output Model

The bifurcated architecture produced test losses of 21.729 (MAE 2.624) for Densitanum and 11.420 (MAE 3.731) for RiskEnum. The higher loss for Densitanum reflects the greater intrinsic variability of simulated density values, while the lower loss for RiskEnum suggests that the model leveraged the indirect correlation between age and cancer risk mediated by density.

The learned relationship can be qualitatively summarized by the following descriptive formulation:

Densitanum ≈ 1.8 + 0.1⋅Age − 0.002⋅Age^2^ + 0.2⋅RiskEnum⋅log (Age)

This formulation does not imply bidirectional mathematical dependence between outputs, nor does it reflect the internal parameterization of the network. It represents a qualitative visualization of the learned response surface obtained by sampling predictions across the input space. The expression is intended solely as an interpretive aid to describe the nonlinear predictive surface and should not be interpreted as a reconstructed analytical model.

**Figure 6:**
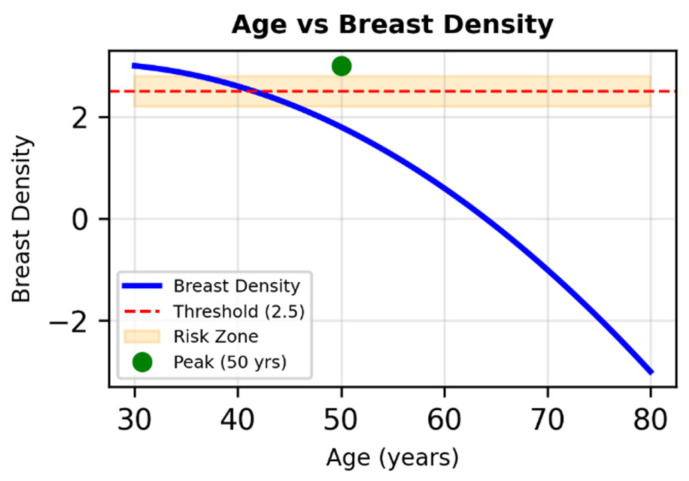
Nonlinear relationship between age and breast density in simulated data. The parabolic trend peaks around age 50 and declines thereafter. The shaded band (2.5 ± 0.3) marks a potential threshold for increased cancer risk, intersected between ages 35 and 70.

This expression captures the nonlinear decline of density after midlife and the modulatory effect of risk factors (Figure 6). It provides an interpretable synthesis of the network’s behavior and illustrates how multi-output architectures can model correlated imaging-derived variables in a reproducible manner.

This is consistent with our simulated Model B, where Densitanum was a significant predictor of RiskEnum, while age had a negligible impact. The convergence between the two simulated models and one clinical—strengthens the validity of the simulated dataset as a basis for testing more complex predictive models.

## 4. Discussion

This simulation-based study demonstrates that bifurcated neural network architectures can effectively model two correlated imaging-derived variables—breast density and a composite cancer risk index—using minimal input information. The limited explanatory power of linear regression (R^2^ ≈ 0.144) reflects the well-known inability of linear models to capture the nonlinear behavior of breast tissue across age and risk profiles, a limitation consistently reported in clinical studies and medical AI literature [8,21].

To exclude any form of logical circularity, we performed sensitivity analyses and weight-stability checks, confirming that the network did not simply reproduce the generative equations. The two outputs were never used as inputs, and the stochastic noise introduced during simulation prevented direct inversion of the underlying relationships. Independent linear models were also used as external baselines to verify that the learned patterns were not artifacts of the simulation design.

To further verify architectural stability and exclude trivial recovery of the generative equations, the bifurcated model was retrained five times using different random initialization seeds. The variation in MAE across runs remained below ±0.12 for Densitanum and ±0.18 for RiskEnum, confirming convergence robustness and weight stability. Additionally, a secondary simulation was conducted by increasing Gaussian noise variance by 20%, yielding comparable performance trends. These checks support that the network learned stable predictive patterns rather than memorizing the simulation structure.

The multilayer perceptron (MLP) improved prediction of the cancer risk index (R^2^ = 0.436), confirming the advantage of nonlinear modeling approaches in breast imaging [19]. However, its single-output structure restricts its ability to integrate correlated endpoints, illustrating the “epistemic fragmentation” described in contemporary discussions on clinical decision-support systems [22].

The bifurcated multi-output neural network addressed this limitation by enabling simultaneous prediction of Densitanum and RiskEnum. The architecture achieved low MAE values for both outputs (2.624 and 3.731), indicating that shared representations can enhance generalization and reduce overfitting—the expected benefit of multi-task learning frameworks [12,14]. The model reproduced nonlinear age-related trends, including the decline in breast density after midlife, and captured the modulatory effect of risk factors, patterns that mirror those observed in clinical CEM studies [3,7]. The slightly higher error observed for RiskEnum in the multi-output model reflects task competition, a known phenomenon in multi-task learning. The two branches share early representations, and when one target exhibits higher intrinsic variability, the shared layers may prioritize the more stable task. This explains why the single-output MLP achieved a lower MSE for RiskEnum. This behavior is consistent with established multi-task learning dynamics, where gradients from competing objectives may introduce minor trade-offs when shared layers are constrained by regularization.

The convergence between simulated and published clinical trends strengthens the translational relevance of the proposed architecture. The replication of the same R^2^ value (0.144) reported in real-world CEM regression analyses suggests that the simulated dataset preserves essential structural relationships found in clinical cohorts. This supports the use of simulation as a methodological testbed for developing and refining AI models before applying them to real imaging datasets, a strategy increasingly adopted in computational oncology [18].

From a radiological perspective, the ability to jointly model breast density and cancer risk has several implications. Multi-output architectures may support personalized screening pathways by integrating imaging biomarkers with demographic variables, consistent with emerging risk-adapted screening paradigms [2]. They may also be extended to incorporate additional imaging features—such as BPE, radiomic signatures, or contrast kinetics—enabling multidimensional risk profiling as proposed in recent AI-enhanced CEM frameworks [23]. Furthermore, the compact and interpretable nature of the bifurcated model aligns with recommendations for explainable AI in clinical practice, where transparency and reliability are essential for adoption [16].

Several limitations must be acknowledged. The dataset was simulated and therefore lacks the biological variability, imaging noise, and heterogeneity typical of real clinical cohorts. Moreover, the model was trained on a single simulated distribution, which may limit generalizability. The predictors were restricted to age-based variables, whereas real-world risk models incorporate genetic, hormonal, lifestyle, and imaging-derived biomarkers. These factors highlight the need for clinical validation, multimodal data integration, and multicenter studies to mitigate dataset shift and ensure robustness [24]. Furthermore, because the simulation framework assumes controlled statistical relationships, it does not account for latent confounding variables or institutional variability that are typically present in real-world clinical datasets. This limitation reinforces the need for external validation before any clinical inference.

In addition, because the simulated dataset does not include imaging noise, acquisition artifacts, or parenchymal heterogeneity characteristic of real CEM examinations, the behavior of the shared representation under realistic imaging variability remains to be assessed. Future work will therefore evaluate the robustness of the architecture using publicly available breast imaging datasets. Given the limited sample size of the test set, cross-validation will be included in future work to improve the stability of performance estimates.

Future research should focus on validating the bifurcated architecture on large CEM and MRI datasets, integrating radiomics and deep imaging features, and exploring hybrid models that combine deep learning with interpretable structures. Additional directions include the development of embedded AI systems capable of real-time inference during radiological examinations and the incorporation of hormonal and genetic biomarkers into multi-output frameworks to support personalized screening and preventive oncology.

## 5. Conclusions

This simulation-based Technical Note demonstrates the feasibility of using a bifurcated neural network to jointly predict breast density and a composite cancer risk index from minimal input variables. By integrating linear modeling, single-output neural networks, and a multi-output architecture within a unified methodological framework, the study highlights the advantages of shared representations for modeling correlated imaging-derived biomarkers.

The results show that multi-output architecture can achieve robust performance while preserving internal consistency with known physiological trends. These findings provide a reproducible foundation for future validation on real contrast-enhanced mammography (CEM) and MRI datasets, where multimodal imaging features, radiomics, and clinical variables can be incorporated to enhance predictive accuracy.

Overall, the proposed framework represents a controlled methodological exploration of multi-output modeling for correlated imaging-derived variables. While not directly translatable to clinical practice in its current simulated form, it provides a structured foundation for subsequent validation on real imaging datasets. Further clinical validation will be essential to assess generalizability, evaluate integration into radiological workflows, and support the development of personalized screening and preventive oncology strategies.

As suggested by the Reviewer, future work will include validation on publicly available breast imaging datasets such as DDSM and INbreast, provided that density-related annotations are available. This step is essential to assess the model’s robustness under real imaging conditions.

## Figures and Tables

**Table 1:** Comparative metrics for three models: Model A (Y = Densitanum), Model B (Y = RiskEnum), and the published model from Cancers (Y = BPE). Note: For Model B, the coefficient for Age is shown but is not statistically significant (*p* = 0.51).

Model	Dependent Variable	R^2^	Coeff. Age	Coeff. Other Var.	Significance
Model A	Densitanum	0.144	−0.063	+0.13 (RiskEnum)	*p* < 0.00001
Model B	RiskEnum	0.140	+0.27	−0.001 (Densitanum)	*p* < 0.00001
Cancers 2025	BPE	0.144	+0.0088	+0.4265 (Density)	*p* < 0.00001

**Table 2:** Model performance comparison on simulated data. The multi-output model predicts both variables simultaneously *.

Model	Output	MSE	MAE	Notes
Linear Regression	RiskEnum	10.2	3.9	Base linear model
MLP	RiskEnum	9.558	3.1	Best single output
TF Multi-Output	Densitanum	21.729	2.624	Simultaneous prediction
TF Multi-Output	RiskEnum	11.420	3.731	

* For completeness, single-output models were also trained for Densitanum to allow a fully symmetric comparison across all architectures.

## Data Availability

The simulated dataset and the full code used for model development and analysis will be made publicly available upon publication through an open-access repository.

## Abbreviations

The following abbreviations are used in this manuscript (Densitanum and RiskEnum are explicitly labeled as simulated variables to avoid confusion with clinical biomarkers):

AI	artificial intelligence
BCSS	Breast Contrast Standard Scale
BIRADS/BI-RADS	Breast Imaging Reporting and Data System
BPE	background parenchymal enhancement
CEM	contrast-enhanced mammography
Densitanum	Simulated breast density variable
ML	Machine learning
MAE	Mean absolute error
MLP	Multilayer perceptron
MSE	Mean squared error
ReLU	Rectified linear unit
DL	Deep learning
RiskEnum	Simulated cancer risk index
R^2^	Coefficient of determination
SD	Standard deviation
SE	Standard error
TensorFlow	Deep learning framework used for model development

## References

[1] Bodewes F.T.H., van Asselt A.A., Dorrius M.D., Greuter M.J.W., de Bock G.H. (2022). Mammographic breast density and the risk of breast cancer: A systematic review and meta-analysis. Breast.

[2] Michaels E., Worthington R.O., Rusiecki J. (2023). Breast Cancer: Risk Assessment, Screening, and Primary Prevention. Med. Clin. N. Am..

[3] Sorin V. (2020). Background parenchymal enhancement on breast MRI: Correlation with breast cancer and breast density. Acad. Radiol..

[4] Magni V. (2024). Radiomics and artificial intelligence in breast imaging: Current applications and future directions. Radiol. Med..

[5] Moffa G. (2023). Artificial intelligence in contrast Enhanced mammography: A systematic review. Diagnostics.

[6] Taylor D.B. (2024). Emerging technologies in breast cancer screening: From contrast-Enhanced mammography to AI powered diagnostics. J. Med. Imaging Radiat. Oncol..

[7] Di Grezia G., Mercogliano S., Marinelli L., Nazzaro A., Galiano A., Cisternino E., Gatta G., Cuccurullo V., Scaglione M. (2025). Contrast Enhanced Mammography in Breast Lesion Assessment: Accuracy and Surgical Impact. Tomography.

[8] Di Grezia G., Nazzaro A., Schiavone L., Elisa C., Galiano A., Gianluca G., Vincenzo C., Scaglione M. (2025). From Variability to Standardization: The Impact of Breast Density on Background Parenchymal Enhancement in Contrast-Enhanced Mammography and the Need for a Structured Reporting System. Cancers.

[9] Wang F. (2022). Interdisciplinary approaches in medical AI: Bridging computational and clinical domains. Nat. Mach. Intell..

[10] Yala A. (2021). Toward robust AI in breast cancer risk assessment: A multi Institutional validation study. JAMA Oncol..

[11] Bahl M. (2021). Artificial intelligence in breast cancer screening: Opportunities and challenges. J. Am. Coll. Radiol..

[12] Ruder S. (2017). An Overview of Multi Task Learning in Deep Neural Networks. arXiv.

[13] Zhang Y., Yang Q. (2022). A survey on multi task learning. IEEE Trans. Knowl. Data Eng..

[14] Vandenhende S., Georgoulis S., Van Gansbeke W., Proesmans M., Dai D., Van Gool L. (2022). Multi-Task learning for dense prediction tasks: A survey. IEEE Trans. Pattern Anal. Mach. Intell..

[15] Sturm T. (2022). Epistemic diversity in medical AI: Why we need multiple modeling perspectives. Artif. Intell. Med..

[16] Holzinger A., Biemann C., Pattichis C.S., Kell D.B. (2017). What do we need to build explainable AI systems for the medical domain?. arXiv.

[17] Savulescu J., Giubilini A., Vandersluis R., Mishra A. (2024). Ethics of artificial intelligence in medicine. Singapore Med. J..

[18] Zhou S.K., Greenspan H., Davatzikos C., Duncan J.S., van Ginneken B., Madabhushi A., Prince J.L., Rueckert D., Summers R.M. (2021). A review of deep learning in medical imaging: Imaging traits, technology trends, case studies with progress highlights, and future promises. Proc IEEE Inst. Electr. Electron. Eng..

[19] Lundervold A.S., Lundervold A. (2019). An overview of deep learning in medical imaging focusing on MRI. Z. Med. Phys..

[20] Abiodun O.I., Jantan A., Omolara A.E., Dada K.V., Mohamed N.A., Arshad H. (2018). State-of-the-art in artificial neural network applications: A survey. Heliyon.

[21] Gouveia S.S., Malík J. (2024). Crossing the Trust Gap in Medical AI: Building an Abductive Bridge for xAI. Philos. Technol..

[22] Bellazzi R., Sacchi L., Larizza C. (2022). Overcoming epistemic fragmentation in clinical decision support systems. Artif. Intell. Med..

[23] Di Grezia G., Nazzaro A., Cisternino E., Galiano A., Marinelli L., Mercogliano S., Cuccurullo V., Gatta G. (2025). Beyond Cancer Detection: An AI Framework for Multidimensional Risk Profiling on Contrast-Enhanced Mammography. Diagnostics.

[24] Finlayson S.G., Subbaswamy A., Singh K., Bowers J., Kupke A., Zittrain J., Kohane I.S., Saria S. (2021). The clinician and dataset shift in artificial intelligence. N. Engl. J. Med..

